# The development of new research methods for the valuation of EQ-5D-5L

**DOI:** 10.1007/s10198-013-0502-3

**Published:** 2013-07-31

**Authors:** Nancy J. Devlin, Paul F. M. Krabbe

**Affiliations:** 1 Office of Health Economics, 105 Victoria Street, London, SW1E 6QT UK; 2 Department of Epidemiology, University Medical Center Groningen, University of Groningen, P.O. Box 30.001, 9700 RB Groningen, The Netherlands

The EQ-5D is arguably now the most well-known and commonly used generic measure of health status internationally. It is available in 169 languages, with applications in clinical, cost-effectiveness and population health studies, as well as (more recently) its routine use by health-care systems. A key feature of the EQ-5D is the availability of ‘value sets’ to weight the EQ-5D health states reported by patients and populations. These value sets provide, for each of the 243 health states described by the EQ-5D, a value (‘utility’) on a scale anchored at 1 (full health) and 0 (dead), reflecting the preferences of the general public, which can be used to estimate quality-adjusted life years (QALYs). These value sets are widely used in the analysis of EQ-5D data and inform a wide range of resource allocation decisions. Value sets for the 3-level version of the EQ-5D (3L) are available in 18 countries and are generally regarded as a credible basis for decision making—for example, the UK value set reported by [1] is recommended by NICE for use in its health technology appraisal process [2]. However, these value sets were largely the result of locally led researcher initiatives. The EuroQol Group never developed or promulgated a formal protocol for the conduct of EQ-5D valuation studies, with the result that value sets studies around the world were undertaken using somewhat inconsistent methods for eliciting, analysing and modelling preferences data.

As use of the EQ-5D has become more common over the last few decades, a growing body of evidence has developed on both its merits and limitations as a health-status measure. Whilst in many applications the EQ-5D has been shown to be a valid and reliable measure of patient health, it has also been argued that in some contexts the three-level version of the EQ-5D may lack sensitivity or fail to capture important aspects of health in certain disease areas. To address this issue, the EuroQol Group has undertaken an ambitious research and development programme aimed at the development of a more sensitive health-status measurement instrument. One of the recent developments was a five-level version, the EQ-5D-5L [3]. An interim set of values for the EQ-5D-5L is available from a cross-over study [4], allowing values for EQ-D-5L states to be assigned from the existing EQ-5D (3L) value sets. But such methods have their limitations—and ultimately decision makers will require EQ-5D-5L value sets that reflect the preferences of the general public over both the dimensions and the expanded levels of the new instrument.

The need to produce value sets to accompany the EQ-5D-5L therefore presented an opportunity to both advance the methods for health-state valuation and develop an agreed protocol to be followed by all countries aiming to produce an EQ-5D-5L value set. In anticipation of the requirement for value sets to accompany the new EQ-5D-5L, the EuroQol Group initiated a programme of methodological research, aimed at overcoming well-known problems and limitations with the time trade-off (TTO) approach that had been used in valuing the three-level version of the EQ-5D and testing the use of discrete choice (DC) methods to value EQ-5D-5L. The conventional approach to TTO is known to have some important problems, particularly relating to the way values are obtained for health states considered to be worse than dead (i.e., values <0). For example, the conventional TTO uses conceptually different approaches to the valuation of states better than dead and worse than dead, resulting in arbitrarily large negative values. Traditionally, this has been redressed by a transformation of the negative values to a range with a minimum of −1. To address the issues, the EuroQol Group initiated research to develop new methods for TTO, resulting in the identification of two potential alternative approaches—the ‘lead-time’ and ‘lag- time’ TTO.

In addition to improving and testing valuation methods, there are additional challenges posed by the valuation of EQ-5D-5L compared to the EQ-5D-3L: most obviously, there are more states to value (3,125 as opposed to 243). More importantly, the more subtle semantic differences between the levels (for example, between ‘severe’ and ‘extreme’ problems, levels 4 and 5, respectively) mean that it is potentially more difficult for participants in valuation tasks to differentiate between the states they are asked to consider. It was therefore important to test and evaluate the preferences data that would be produced for EQ-5D-5L states using the methods under consideration.

The articles in this issue of the *European Journal of Health Economics* report in detail on an ambitious programme of research undertaken by the EuroQol Group to achieve those aims. Specifically, these articles draw together the findings from a multi-country pilot study to test an initial version of the study protocol and related experimentation with aspects of the methods in that protocol. This included experiments regarding a number of aspects of the TTO, including a comparison of the lead and lag-time TTO (Augustovski et al.), a comparison of both those approaches with the conventional TTO (Versteegh et al.), alternative ways of displaying the lead-time TTO (Luo et al.) and experiments with alternative lengths of time in both full health and the state being valued (Versteegh et al. and Luo et al.). All the studies in the multi-country pilot included both TTO and DC methods; the article by Ramos-Goni et al. reports on the use of DC data to value EQ-5D-5L states and on the potential to combine DC with direct comparisons of each state with ‘dead’, as a means of anchoring to 0. Finally, this series of papers ends with two articles that each address related issues. Based on in depth study of the literature, Attema et al. present an overview of the various alternative specifications that exist for the TTO. In the last article, Shah et al. present results of a separate investigation on the characteristics and the quality of the data generated in settings for administering TTO tasks if based on computer-assisted interviews that were interviewer-led (one-to-one) versus the same TTO tasks in a self-complete setting (with group assistance).

The research reported here helped to inform decisions about the final international protocol to be used in studies to produce value sets for the EQ-5D-5L [5]. For example, a key finding of the work described in the following articles, and summarised in the article by Shah et al. in this issue, was the clear importance of administering TTO tasks in face-to-face interviews—alternative modes of data collection, such as allowing respondents to self-complete TTO tasks in group sessions, compromised the credibility of the data. Additionally, whilst the lead-time TTO has advantages over the conventional approach in eliciting values <0, earlier research had highlighted concerns about the choice of the length of the lead time exerting a framing effect on values [6]. A ‘composite TTO’ approach appeared to provide a promising alternative, by using the conventional approach to the elicitation of values >0, and using the lead time approach for values <0. This was tested for the first time in The Netherlands, and the results reported in this issue showed that the method produced highly credible data (Janssen et al.).

The final protocol, described in detail in Oppe et al. [5], includes software for use in computer-assisted personal interviews, an interviewer script, interviewer training materials, guidance to researchers and tools for data analysis. The protocol employs a combination of methods—both the composite TTO and DC—for the valuation of EQ-5D-5L states. The articles constitute an important body of work that helped refine and improve the methods in the protocol and will now be rolled out in a series of pivotal studies internationally. Value sets using these methods will be reported later this year in The Netherlands, Spain, England, Canada and China. Further studies are now underway or planned in Japan, Taiwan, South Korea, Germany, Uruguay, Thailand, Hong Kong and Singapore, with interest expressed also from others. We confidently expect there to be many more EQ-5D-5L value sets available than was the case for EQ-5D-3L, ensuring that appropriate value sets will be available for use in local applications of the instrument.

For the first time, the EuroQol Group has a fully documented research protocol. This will ensure that studies are undertaken to a high standard, using a consistent study design and methods for collecting health-state values. Ultimately, this will also create a unique opportunity for international comparisons of values for EQ-5D-5L. Of course, inevitably there are many remaining methodological issues, which can and will be addressed in ongoing research—but the international protocol represents a significant achievement for the EuroQol Group, and a step forward in the use and application of the EQ-5D-5L.
